# Impact of *NLRP1* Met1154Val and *IL1B* variants on gestational malaria: an unexplored role of NLRP1 in inflammasome activation by *Plasmodium* spp.

**DOI:** 10.1002/path.6471

**Published:** 2025-09-09

**Authors:** Vinicius NC Leal, Jessica A Ribeiro, Leticia Girardi Marra, Alexandre TT Rio, Edione C Reis, Gerhard Wunderlich, Jamille G Dombrowski, Claudio RF Marinho, Alessandra Pontillo

**Affiliations:** ^1^ Departamento de Imunologia Instituto de Ciências Biomédicas, Universidade de São Paulo (ICB/USP) São Paulo Brazil; ^2^ Departamento de Parasitologia Instituto de Ciências Biomédicas, Universidade de São Paulo (ICB/USP) São Paulo Brazil

**Keywords:** inflammasome, NLRP1, gestational malaria, *Plasmodium falciparum*, *Plasmodium vivax*, trophoblasts

## Abstract

We hypothesized that variants in inflammasome‐related genes could influence susceptibility to gestational malaria (GM). To test this, we conducted an association study in a cohort of pregnant women from a malaria‐endemic region in northern Brazil, assessing whether specific functional single nucleotide variants (SNVs) in inflammasome genes affect (1) the response to *Plasmodium* infection and (2) the development of placental malaria. Our findings revealed that the *NLRP1* p.Met1154Val variant was associated with a protective effect against *Plasmodium* infection. Moreover, *IL1B* SNVs appeared more prevalent in severe cases. Additionally, multivariate analyses incorporating placental blood cytokines, growth factors, and immunohistochemical features revealed that the *NLRP1* p.Met1154Val variant correlated with a healthier placental state, highlighting a potential protective role of the NLRP1 inflammasome in GM. For the first time, we showed that infected red blood cells induce NLRP1‐ and caspase‐1‐dependent pyroptosis in BeWo trophoblast cells, identifying a novel inflammasome pathway involved in GM pathogenesis. Our study identifies a genetic variant underlying NLRP1 contribution to GM and suggests that NLRP1 may be an under‐explored inflammasome receptor in malaria and infected erythrocytes' sensing. © 2025 The Author(s). *The Journal of Pathology* published by John Wiley & Sons Ltd on behalf of The Pathological Society of Great Britain and Ireland.

## Introduction

Malaria in pregnancy poses serious risks to both mother and fetus, leading to complications such as miscarriage, stillbirth, and low birth weight. Placental malaria (PM), a severe form of gestational malaria (GM), occurs in 20%–50% of cases, due to immunological dysregulation, *Plasmodium*‐infected red blood cell (iRBC) sequestration within the placenta, and consequent severe inflammation, which finally lead to placental dysfunction [1, 2, 3].

Several factors, including young maternal age, first pregnancy, *Plasmodium* species (*P. falciparum* causing the most severe effects), and host genetics, influence GM susceptibility and severity [1, 2, 3, 4]. The pathogenesis of GM still needs to be fully elucidated, and it can present different prognoses in different women [1, 4]. Understanding inter‐patient heterogeneity is critical for identifying diverse immunological pathways involved in host–pathogen interactions and, consequently, for improving treatment and follow‐up care for pregnant women.

We recently demonstrated that inflammasome‐dependent IL‐1β release plays a key role in pregnancy outcome in a mouse model of GM. The high amount of IL‐1β in the placenta correlated with reduced intrauterine fetal growth. iRBCs induced caspase‐1 activation and IL‐1β release in macrophages and trophoblast cells [5]. Human trophoblasts and placenta‐associated monocytes/macrophages express inflammasome and many of its receptors [6, 7] and thus can respond to *Plasmodium‐*associated molecular patterns and/or to iRBCs or damaged tissue debris [8]. Nlrp3 and Aim2, previously reported as key inflammasome receptors in murine models of malaria by detecting hemozoin [9], a byproduct of iRBC metabolism, and *Plasmodium* DNA [10] respectively, contribute to the severity of our model of GM [5].

In humans, functional single nucleotide variants (SNVs) in inflammasome receptors or the *IL1B* gene affect the level of inflammasome activation and can contribute to heterogeneity in individual responses to pathogens [11]. We previously demonstrated that *NLRP1* variants were associated with severe *vivax* malaria [12]. Although several previous studies have demonstrated the role of NLRP1 as a sensor in viral infections [13], there is currently no evidence of NLRP1 involvement in immune response against *Plasmodium* spp. and little is known about its role in other protozoan diseases [8]. NLRP1 has been identified as crucial in *Toxoplasma gondii* infection [14, 15, 16, 17], indicating a potential role in the innate immune response against protozoan parasites.

NLRP1 is expressed in the placenta (http://www.proteinatlas.org), and specifically in trophoblasts [7], where it has been suggested to contribute to oxidative stress response [18, 19], one of the hallmarks of dysfunctional placenta [1, 2, 3]. *T. gondii* has been suggested to induce pyroptosis in the placenta by inducing reactive oxygen species (ROS) and inflammasome activation partially depending on NLRP1 [16].

Additionally, *NLRP1* presents some variants with high frequency (30%–50%) that have previously been associated with infections [11] and pre‐eclampsia [20], making it a reasonable target for a gestational infection disease. The distribution of these variants shows racial disparity, with a frequency of 30%–50% in European and African regions where malaria was or still is endemic, and of 3%–19% in the Asian populations (https://ensembl.org).

In this study, we genotyped a cohort of pregnant women from a malaria‐endemic region of Brazil and found that the *NLRP1* Met1154Val variant protects against GM and correlates with a healthier placental state, highlighting a potential protective role of the NLRP1 inflammasome in GM. Moreover, we confirmed the impact of *IL1B* SNVs, not only in *vivax* malaria but also in GM. As a proof of concept, we demonstrated that iRBCs can activate the NLRP1 inflammasome in BeWo trophoblasts. Our findings demonstrate the contribution of *NLRP1* and *IL1B* SNVs in GM and suggest a novel mechanism for GM pathogenesis.

## Materials and methods

### Ethics approval

The study was approved by the ethics committees of the University of São Paulo and the Federal University of Acre (Plataforma Brasil, CAAE: 03930812.8.0000.5467 and 03930812.8.3001.5010, respectively). All study participants or their legal guardians (if minors) provided written informed consent.

### Patient cohort

453 pregnant women were recruited from the Hospital da Mulher e da Criança in Juruá (AC, Brazil), an endemic area for malaria. GM was confirmed in 282 women using molecular diagnostic testing of blood samples. 91 out of 282 infected women were diagnosed with PM, defined by the presence of parasites (predominantly mixed or *falciparum* infection) and hemozoin deposition in the placenta [21, 22]. Exclusion criteria included smoking, drug use, alcohol consumption, HIV, HBV, syphilis, and toxoplasmosis during pregnancy. Cohort information has been detailed in [22] and is briefly summarized in Table 1.

**Table 1:** Characteristics of women with and without gestational malaria.

	Gestational malaria (GM) (*n* = 191)	Placental malaria (PM) (*n* = 91)	Non‐infected pregnant women (NI) (*n* = 171)
Age, years	21 (17–27)	22.24 (13.0–38.0)	23 (19.0–28.0)
Ethnicity (W/M/B), *n*	35/220/27	10/74/7	22/135/14
Parity (one/more), *n*	114/168	37/54	81/90
Leukocytes (cells/μl), mean (min–max)	13.06 (3–29.6)	12.56 (5.4–24.2)	13.1 (5.05–25.60)
Platelets (cells/μl), mean (min–max)	190.97 (42–507)	170.39 (42.0–275.0)	205,73 (111–325)
Anemia (yes), *n* (%)	87 (31)	37 (41)	28 (16)
Hemoglobin (g/dl), mean (min–max)	11.4 (5–15.3)	11.23 (5.40–14.10)	12 (8.7–14.8)
Infection			
Parasitemia (parasites/ml), mean (min–max)	3,879.64 (0.50–75,520.0)	4,490.12 (1.37–61,120.00)	–
Malaria episodes (one/more), *n*	123/159	30/61	–
Fever (>37 °C), *n* (%)	63 (22)	19 (21)	–
Type (*falciparum*/*vivax*/mixed), *n* (%)	74 (26)/141 (50)/67 (24)	37 (41)/11 (12)/43 (47)	–
Placenta hemozoin (high/low), *n*	33/51	33/50	–
Placental features			
Placental syncytial nuclear aggregates, mean (min–max)	16.32 (1–51)	16.4 (1–47)	13.89 (3.0–34.0)
Placental necrosis, mean (min–max)	8.16 (0.87–26.29)	8.0 (0.96–26.29)	7.55 (0.42–26.79)
Placental fibrin, mean (min–max)	2.34 (0.83–3.83)	2.28 (0.83–3.75)	2.09 (0.92–3.67)
Placental vascularity, CD31^+^, mean (min–max)	4.09 (2.0–7.9)	4.05 (2.20–6.10)	4.01 (2.0–6.0)
Placental leukocytes, CD45^+^, mean (min–max)	22.94 (3.0–75.0)	23.80 (3.0–75.0)	17.59 (2.0–68.0)
Placental monocytes, CD68^+^, mean (min–max)	9.57 (1.0–50.0)	10.04 (1.0–50.0)	4.96 (1.0–22.0)

*Note*: W/M/B, white/mixed/black.

### DNA samples and SNV genotyping

Maternal DNA was previously extracted from whole blood using standard methods and stored in a biorepository (CAAE: 03930812.8.0000.5467; Of.028.11). SNVs in *NLRP1* (rs12150220, rs11651270), *NLRP3* (rs10754558, rs35829419), *IL1B* (rs16944, rs1143634), *IL18* (rs5744256), *P2RX7* (rs2230911), and *DPP9* (rs12610495) genes were selected based on literature and our previous results [11, 12], and genotyping was performed using allele‐specific TaqMan assays (Applied Biosystems, Thermo Fisher Scientific, Foster City, CA, USA) (catalog numbers are provided in supplementary material, Table S1) and qPCR on QuantStudio 3.0 equipment (Applied Biosystems). Allelic discrimination was performed using the QuantStudio 3.0 software (Applied Biosystems). The genotyping concordance rate was 99.5% across replicates.

### RBC/iRBC preparation

*P. falciparum‐*infected red blood cells (iRBCs) (5%–10% parasitemia) were counted and submitted to ten freeze–thaw cycles to generate the cell lysate. Non‐infected RBCs (RBCs) were quantified and submitted to the same lysis process.

### BeWo cell culture and treatment

BeWo cells were cultured in RPMI‐10% FBS (Gibco, Thermo Fisher Scientific, Grand Island, NY, USA), plated in 96‐ or 24‐well plates, and treated with non‐infected RBCs at a ratio of 40:1 (RBC:BeWo) and with iRBCs at a ratio of 10:1, 20:1, and 40:1 for 24–48 h [5]. Alternatively, priming with bacterial lipopolysaccharide (LPS; 10 or 100 ng/ml) (Sigma‐Aldrich, Millipore/Merck, St. Louis, MO, USA) or TNF (5, 10, or 20 ng/ml) (PeproTech Inc., Thermo Fisher Scientific, Rocky Hill, NJ, USA) was carried out before RBC/iRBC treatment.

In some experiments, cells were treated with the DPP‐9 inhibitor talabostat (Val‐boroPro/VbP; 10 μm) (Tocris Bioscience, Bristol, UK) [23] or incubated with the selective NLRP3 inhibitor MCC950 (Invivogen, San Diego, CA, USA) [24], or the dual NLRP1 and NLRP3 inhibitor ADS032 (MedChemExpress LLC, Monmouth Junction, NJ, USA) [25] 60 min before RBC/iRBC treatment.

### Gene expression analysis

Total cellular RNA was extracted using the RNAqueous mini kit (Ambion Inc., Thermo Fisher Scientific, Austin, TX, USA), followed by reverse transcription to cDNA using a SuperScript III Reverse Transcriptase kit (Invitrogen, Thermo Fisher Scientific, Carlsbad, CA, USA). Relative gene expression was assessed by qPCR using gene‐specific Taqman Assays (Applied Biosystems) (catalog numbers are provided in supplementary material, Table S1). Data are presented as basal expression normalized to the *GAPDH* endogenous control. Triplicates were used for all conditions and for each independent experiment. The data concordance rate was 99.5% across replicates.

### Flow cytometry analysis of activated caspase‐1

Caspase‐1 activation was measured using the FAM‐FLICA Caspase‐1 Assay Kit and flow cytometry following the manufacturer’ s protocol (Immunochemistry Technologies, Bloomington, MN, USA). In brief, 0.5 × 10^6^ cells were incubated with the FAM‐FLICA probe for 30 min and then with LIVE/DEAD Cell Stain (Invitrogen). Samples were analyzed using the BD Canto II flow cytometer [Becton, Dickinson and Company (BD), Franklin Lakes, NJ, USA] and FlowJo_V10 software (https://www.flowjo.com/). Data are presented as percentage of FAM‐FLICA‐positive cells.

### Dosage of IL‐1β and lactate dehydrogenase (LDH)

The release of IL‐1β and LDH was measured in culture supernatants using colorimetric assays (R&D Biosystems, Minneapolis, MN, USA; Thermo Fisher Scientific). Triplicates were used for all conditions and for each independent experiment. The data concordance rate was 99.5% across replicates. Data are presented as pg/ml (IL‐1β) and percentage of LDH release versus Triton X‐100‐treated cells (= 100%), respectively.

### Immunofluorescence assay

Cells were cultured on coverslips, treated as above, stained with anti‐NLRP1 antibody (1:200; Abcam, Cambridge, UK) and appropriate secondary antibody together with the nuclear dye DAPI (Thermo Fisher Scientific), and finally observed using fluorescence microscopy (Axio Vert.A1; Zeiss, Oberkochen, Germany). Quantification of NLRP1 oligomers was performed using ImageJ software (NIH, Bethesda, MD, USA), as previously reported [26].

### Data analysis

Multivariate analysis and the general linear model (GLM) were used to analyze the SNVs’ association with clinical or laboratory variables using R‐program software (http://www.r-project.org) and the ‘SNPassoc’ package [27]. Confounding variables were selected based on GLM analysis of the principal variables and used to adjust the SNV distribution analysis [adjusted *p* value (*p* adj)] as specified for each analysis step. To reduce the skewness of the data, variables with a wide range of values were log‐transformed to make them more suitable for analysis. A formal Bonferroni correction for the number of independent SNPs analyzed would require a significance threshold of *p* = 0.01 (*p*0/*n*, *p*0 = 0.05, *n* = 5 genes/loci). GraphPad Prism software (GraphPad Software Inc., San Diego, CA, USA) was used for data analysis and graphing.

## Results

To explore the contribution of inflammasome genetics in GM, we genotyped selected functional SNVs in the GM cohort from a malaria‐endemic area of the Brazilian Amazon (Table 1). All the analyzed SNVs were in Hardy–Weinberg equilibrium in the cohort, and their allelic frequencies were similar to those reported in 1000 Genomes Project (https://internationalgenome.org) (supplementary material, Table S2).

**Figure 1:**
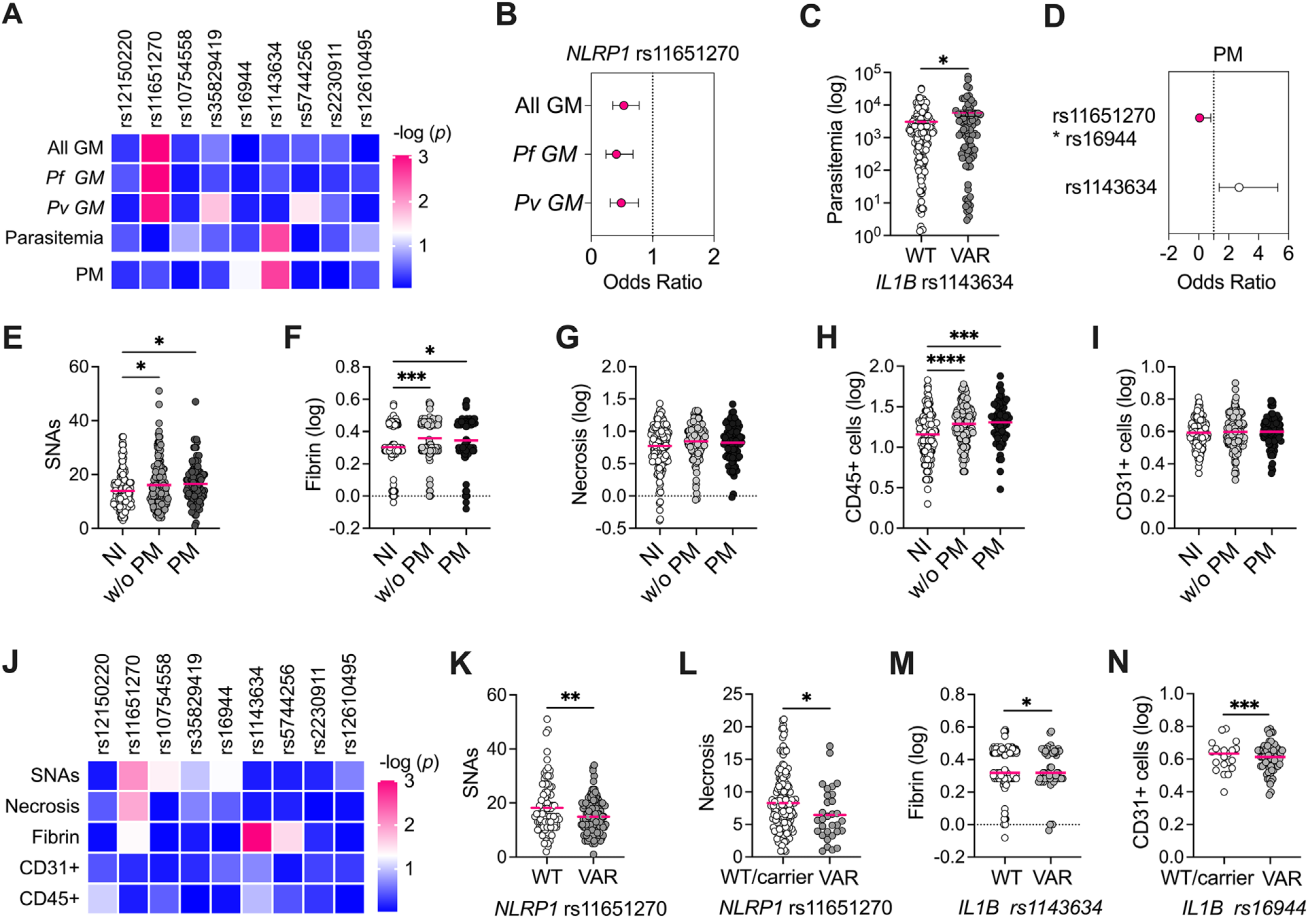
Effect of inflammasome SNVs in the predisposition to gestational malaria. (A) Heatmap of adjusted *p* value calculated for selected inflammasome SNVs in *NLRP1* (rs12150220, rs11651270), *NLRP3* (rs10754558, rs35829419), *IL1B* (rs16944, rs1143634), *IL18* (rs5744256), *P2RX7* (rs2230911), and *DPP9* (rs12610495) genes in case/control association analysis [gestational malaria (GM) versus non‐infected pregnant women (NI)] taking into account either all GM cases, or just *P. falciparum* or *P. vivax* infections (All GM, *Pf* GM, *Pv* GM, respectively). The distribution of SNVs according to parasitemia and to the presence of placental malaria (PM) has also been included in this analysis step and in the heatmap (parasitemia, PM). Age and number of pregnancies are included in the general linear model (GLM) as confounding variables for case/control analysis. Age, number of pregnancies, type of infection (*P. falciparum* or *P. vivax* or mixed), and episodes of malaria are included in the GLM as confounding variables in GM analysis. (B) Forest plot of odds ratio (OR) values and confidence intervals for *NLRP1* rs11651270 SNV in case/control association analysis (All GM, *Pf* GM, *Pv* GM). (C) Distribution of parasitemia values according to *IL1B* rs1143634 SNV. The difference between women who do not carry the polymorphic allele (WT, G/G) and who carry the polymorphic allele (VAR, G/A or A/A) was calculated using the GLM adjusted for age, number of pregnancies, type of infection (*P. falciparum* or *P. vivax* or mixed), and episodes of malaria. (D) Forest plot of odds ratio (OR) values and confidence intervals for the rs1143634 variant and the SNV combination *NLRP1* rs11651270/IL1B rs16944 in GM association analysis for the presence of PM. (E–I) Placental histologic features [syncytial nuclear aggregates (SNAs), fibrin deposition, necrosis, leukocyte infiltration/CD45^+^ cells, and vascularity/CD31^+^ cells] were analyzed and compared among non‐infected pregnant women and infected women without or with PM (NI, GM w/o PM, and PM, respectively) using a one‐way ANOVA test followed by a multiple comparisons post‐test. (J) Heatmap of adjusted *p* value calculated for inflammasome SNVs in *NLRP1* (rs12150220, rs11651270), *NLRP3* (rs10754558, rs35829419), *IL1B* (rs16944, rs1143634), *IL18* (rs5744256), *P2RX7* (rs2230911), and *DPP9* (rs12610495) genes and dysfunctional placenta features in GM cases. Age, number of pregnancies, type of infection (*P. falciparum* or *P. vivax* or mixed), and episodes of malaria are included in the GLM as confounding variables. (K,L) Syncytial nuclear aggregates (SNAs) and necrosis distribution according to *NLRP1* rs11651270 SNV are shown for GM women. The differences were calculated using the GLM adjusted for age, number of pregnancies, type of infection (*P. falciparum* or *P. vivax* or mixed), and episodes of malaria. (M) Distribution of fibrin deposits according to *IL1B* rs1143634 SNV is shown for GM women. The differences were calculated using the GLM adjusted for age, number of pregnancies, type of infection (*P. falciparum* or *P. vivax* or mixed), and episodes of malaria. (N) Vascularity (CD31^+^ cells) distribution according to *IL1B* rs16944 SNV is shown for PM women. Differences were calculated by the GLM adjusted for age, number of pregnancies, type of infection (*P. falciparum* or *P. vivax* or mixed), and episodes of malaria. *p < 0.05, **p < 0.01, ***p < 0.001, ****p < 0.0001.

Initially, we compared the distribution of SNVs in pregnant women with and without GM (case/control study) using a multivariate test adjusted for confounding variables (age, previous pregnancies) based on the prior linear regression assessment of dependence (supplementary material, Table S3). None of the selected SNVs were found to be associated with risk of GM, except the *NLRP1* p.Met1154Val (rs11651270 A>G) variant (Figure 1A). This variant was more frequently observed in non‐infected women (73.8%) than in women with malaria (59.9%), suggesting a protective effect of this polymorphism against GM (*p* adj = 0.001, OR adj = 0.53; 95% CI 0.35–0.78) (Figure 1B and supplementary material, Table S4). Since our cohort included both *falciparum* and *vivax* malaria, and a group of women was positive for both two parasites (mixed infection), we stratified our analysis according to the type of infection: *falciparum* or *vivax*. In both sub‐groups, the only associated variant was rs11651270 (*p* adj = 0.001 and 0.002, respectively) (Figure 1A,B and supplementary material, Table S5), confirming a protective role against GM, independent of the type of parasite.

In our previous work [12], we observed a significant association between the variant *IL1B* p.Phe105= (rs1143634 C>T; +3954 C>T) and *P. vivax* parasitemia, which seems to be in accordance with the protective role of this cytokine in infection. In this study, we replicated the association with this variant (*p* adj = 0.015) (Figure 1A,C and supplementary material, Table S4), reinforcing that the cytokine is essential in controlling parasite infection.

When the analysis was performed to detect any possible combinatory effect between the SNVs (epistatic analysis) on parasitemia, we observed that *NLRP1* rs11651270 interacts with *IL1B* rs1143634 (*p* = 0.024). The effect of this interaction was demonstrated by the association of the *NLRP1* and *IL1B* SNV combination on parasitemia (*p* adj = 0.016) (supplementary material, Figure S1).

Among women with GM, 91 presented with PM. Multivariate association analysis identified the *IL1B* SNV rs1143634 as a possible risk factor for the development of PM, the polymorphic allele A being more frequent in women with PM than in those without PM (*p* adj = 0.003; OR adj = 2.69). On the other hand, the combination of the gain‐of‐function variants *NLRP1* rs11651270 and *IL1B* rs16944 promotes a protective effect on the development of PM (Figure 1A,D and supplementary material, Table S6).

p.Phe105= (rs1143634 C>T) and the promoter variant −511 G>A (rs16944) have been associated with lower and higher IL‐1β levels, respectively [28, 29]. Therefore, our data show that IL‐1β is a protective factor for PM, especially together with NLRP1.

Syncytial nuclear aggregates (SNAs), fibrin deposits, necrosis, increased leukocyte infiltrate, and decreased vascularity are characteristic features of a dysfunctional placenta [1, 2, 3, 4, 22]. In our cohort, all these characteristics, with the exception of necrosis and vascularity, were more pronounced in GM than in non‐infected (NI) women, however without any significant difference between the PM, and GM without PM (GM w/o PM) groups (Figure 1E–I).

In the next step of our association study, we analyzed the distribution of SNVs according to these different features of the placenta in GM women.

The *NLRP1* rs11651270 variant was significantly more frequent in GM women with low levels of syncytial nuclear aggregates and necrosis within the placenta (*p* adj = 0.003 and 0.028, respectively) (Figure 1J–L and supplementary material, Table S7). The *IL1B* rs1143634 variant was significantly more frequent in GM women with high fibrin deposition (Figure 1J,M and supplementary material, Table S7). The *IL1B* rs16944 variant was significantly more frequent in PM women with low vascularity (expressed as CD31^+^ cells) in the placenta (Figure 1J,N and supplementary material, Table S7).

Altogether, the genetic association findings emphasized the role of IL‐1β in GM as we previously demonstrated [5], and identified NLRP1 as a possible alternative sensor for inflammasome activation in malaria.

**Figure 2:**
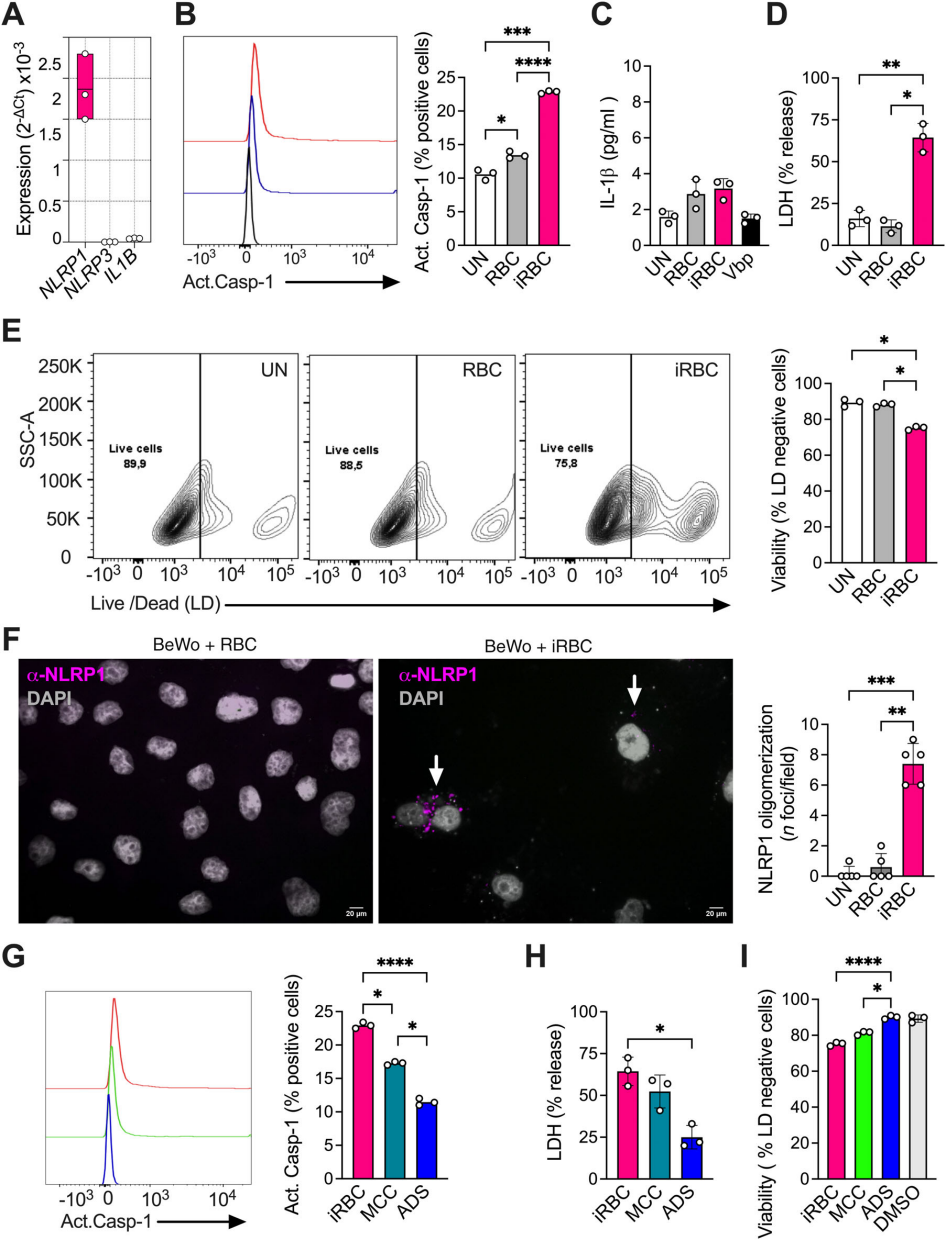
*P. falciparum* iRBCs induce NLRP1 inflammasome‐dependent cell death in BeWo trophoblast cells. (A) Relative expression of *NLRP1*, *NLRP3*, and *IL1B* genes in BeWo trophoblast cells. Data are reported as 2^−∆Ct^, where ∆Ct is obtained by subtracting the Ct value of the housekeeping gene *B2M* from the Ct value of the target gene. The floating bars show the mean and min–max interval of three biological replicates in one experiment. (B) The percentage of activated caspase‐1‐positive BeWo cells in untreated (UN) and treated conditions was analyzed by flow cytometry. An example histogram is shown on the left and a bar graph with mean and scatter dot blots of three experiments on the right. One‐way ANOVA with multiple comparisons post‐test. (C) IL‐1β release was measured by ELISA and expressed as pg/ml in the bar graph, with mean ± SD and scatter dot blot for three experiments. Val‐boroPro (VbP) was used as a control for NLRP1 activation. (D) The percentage of LDH release was measured by colorimetric assay and reported as a bar graph with mean and scatter dot blots for three experiments. Val‐boroPro (VbP) was used as a control for NLRP1 activation. One‐way ANOVA with multiple comparisons post‐test. (E) The percentage of live BeWo cells in untreated and treated conditions was analyzed by flow cytometry. An example dot plot is shown on the left and a bar graph with mean and scatter dot blots of three experiments on the right. One‐way ANOVA with multiple comparisons post‐test. (F) Immunofluorescence staining for NLRP1 in treated and untreated BeWo cells. The panels show representative images (left), and the graph shows the quantification of NLRP1 oligomerization positive cells (right). Scale bars, 20 μm. The mean ± SD represents five visual fields. One‐way ANOVA with multiple comparisons post‐test. (G) The percentage of activated caspase‐1‐positive BeWo cells in untreated and treated conditions was analyzed by flow cytometry. An example histogram is shown on the left and a bar graph with mean and scatter dot blots of three experiments on the right. One‐way ANOVA with multiple comparisons post‐test. (H) The percentage of LDH release was measured by colorimetric assay and reported as a bar graph with mean and scatter dot blots for three experiments. Dimethyl sulfoxide (DMSO) was used as a control for the NLRP3 inhibitor MCC‐950 (MCC) and the NLRP1 and NLRP3 inhibitor ADS‐032 (ADS). One‐way ANOVA with multiple comparisons post‐test. (I) The percentage of live BeWo cells in untreated and treated conditions was analyzed by flow cytometry. Bar graphs with mean and scatter dot blots of three experiments are shown. DMSO was used as a control for MCC‐950 and ADS‐032. One‐way ANOVA with multiple comparisons post‐test. *p < 0.05, **p < 0.01, ***p < 0.001, ****p < 0.0001.

In experimental models of malaria [8] and GM [5], Nlrp3 and Aim2 inflammasomes have been identified as key sensors for *Plasmodium* spp.; however, at that point, NLRP1 had not been taken into account. To validate the genetic association results, and considering the evolutionary differences between rodents and the human *NLRP1* gene [30], we performed a set of experiments with BeWo trophoblast cells as a model for placental *Plasmodium* infection [5]. BeWo cells expressed high levels of *NLRP1* (Figure 2A) compared with *NLRP3*, which was previously investigated in this context [5]. Priming with LPS or TNF did not alter *NLRP1* expression levels (supplementary material, Figure S2). To investigate whether NLRP1 contributes to placental inflammation during GM, we analyzed inflammasome activation in BeWo trophoblast cells at different time points of culture, in the presence of *P. falciparum* iRBCs. This was assessed using a fluorescent probe for activated caspase‐1 combined with flow cytometry analysis, as well as colorimetric assays for LDH release and cytokine quantification.

iRBCs significantly doubled the percentage of BeWo cells positive for activated caspase‐1 compared with untreated or RBC‐treated cells (Figure 2B). The increase in caspase‐1 activation correlated with a significant rise in LDH release, but not IL‐1β release, in iRBC‐treated BeWo cells (Figure 2C,D), along with a significant reduction in cell viability, as observed via flow cytometry (Figure 2E). The effects of iRBC treatment were comparable to those of the known NLRP1 activator, the DPP9 inhibitor talabostat/Val‐boroPro (VbP), in terms of both IL‐1β and LDH release (Figure 2C,D).

**Figure 3:**
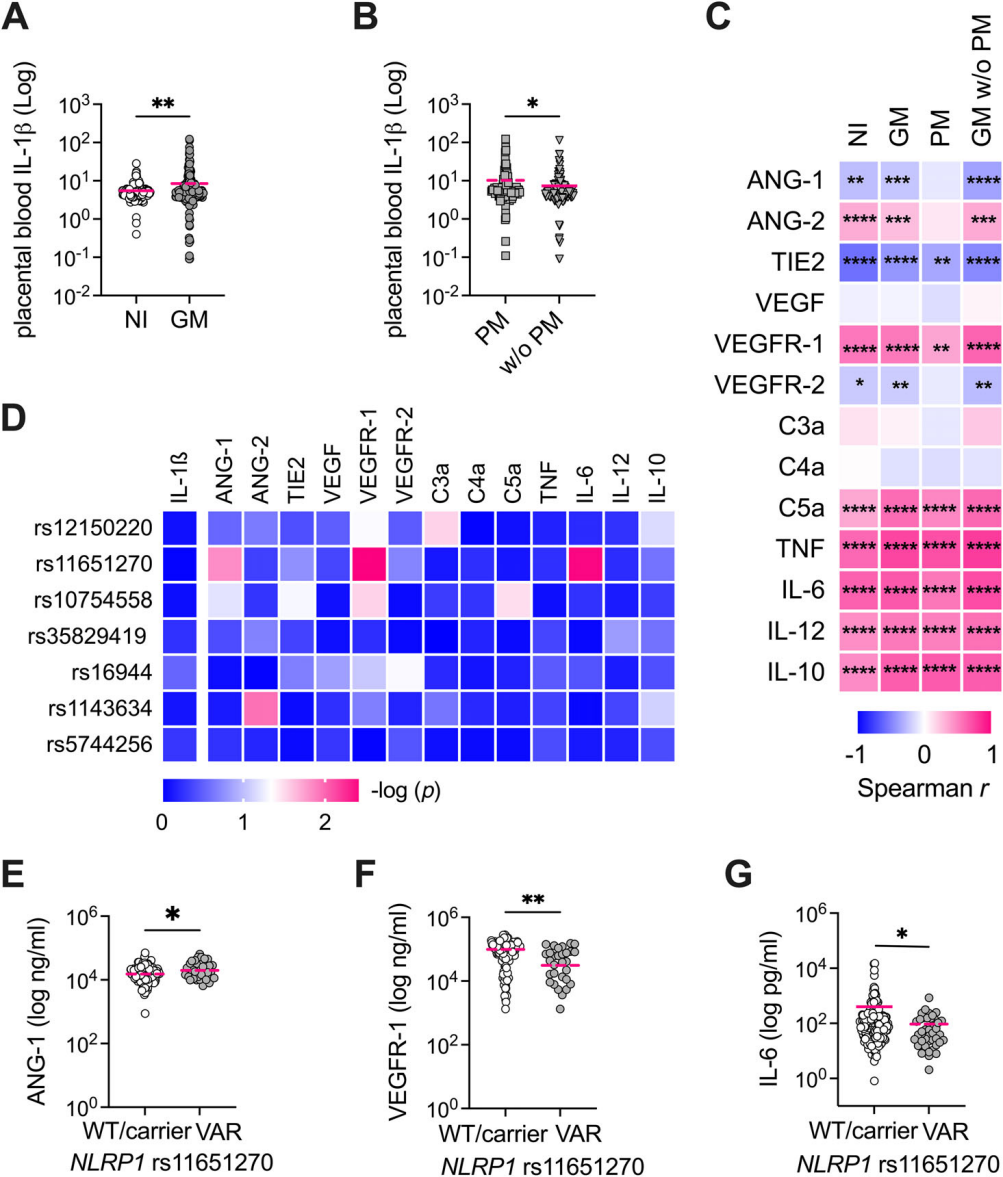
The *NLRP1* rs11651270 variant affects the placental blood levels of IL‐6 and angiogenic factors angiopoietin 1 and VEGFR‐1. (A, B) Placental blood levels of IL‐1β were compared between non‐infected pregnant women (NI) and infected women (GM) (A) and between GM women with or without PM (B) by means of an unpaired *t‐*test. (C) Heatmap of Spearman *r* values calculated for the correlation between IL‐1β levels and other placental blood factors in non‐infected pregnant women (NI), infected women (GM), and GM women with or without (w/o) PM. (D) Heatmap of adjusted *p* value calculated for inflammasome SNVs in *NLRP1* (rs12150220, rs11651270), *NLRP3* (rs10754558, rs35829419), *IL1B* (rs16944, rs1143634), and *IL18* (rs5744256) genes, and placental blood factors taking into account the GM women. Age, number of pregnancies, type of infection, and malaria episodes are included in the general linear model (GLM) as confounding variables. (E–G) Distribution of placental blood factors according to *NLRP1* rs11651270 SNV are shown for GM women. The differences were calculated by the GLM adjusted for age, number of pregnancies, type of infection, and malaria episodes. *p < 0.05, **p < 0.01, ***p < 0.001, ****p < 0.0001.

To assess the role of NLRP1 in inflammasome activation, we examined NLRP1‐positive oligomers. Treatment with iRBCs, but not RBCs, induced a significant increase in NLRP1‐positive oligomers in BeWo cells (Figure 3F), suggesting that iRBCs activate caspase‐1 through NLRP1. Since a specific NLRP1 inhibitor is not yet available, we repeated the *in vitro* assays using ADS‐032, an inhibitor of both NLRP1 and NLRP3 [25], as well as MCC‐950, a known NLRP3 inhibitor [24]. Treatment with ADS‐032 significantly reduced caspase‐1 activation (Figure 2G) and cell death, as measured by LDH release (Figure 2H) and cell viability via flow cytometry (Figure 2I).

Our pyroptosis assays are consistent with previous findings in human trophoblasts, where pyroptosis has been described as a pathogenic mechanism in gestational diseases, namely pre‐eclampsia [31] and obstetric antiphospholipid syndrome [32], even though NLRP1 has not been considered as the initial sensor.

To identify the possible mechanism linking the associated inflammasome variants with the outcomes of malaria infection during pregnancy, we performed an association analysis, taking into account available data from the measurements of cytokines, anaphylatoxins, and angiogenetic and growth factors in the placental blood. IL‐1β levels were increased in GM compared with NI placental blood (Figure 3A), and in GM with PM compared with GM without PM (Figure 3B). These levels of IL‐1β correlated positively with cytokines (TNF, IL‐6, IL‐12, and IL‐10), and anaphylatoxin C5a, and with vascular endothelial growth factor receptor 1 (VEGFR‐1) in all the groups. IL‐1β correlated inversely with angiopoietin‐1 receptor (TIE2) in all the groups, and with vascular endothelial growth factor receptor 2 (VEGFR‐2) and angiopoietin‐1 (ANG‐1) in all the groups except the PM one (Figure 3C) (supplementary material, Table S8).

When we performed multivariate analysis of placental blood factor levels according to SNV genotypes, we observed an intriguing association for the *NLRP1* rs11651270 variant in GM women (Figure 3D). The polymorphic genotype C/C (Val1154) was significantly associated with high levels of ANG‐1 (Figure 3E) and low levels of VEGFR‐1 (Figure 3F). Additionally, we observed that rs11651270 was more frequent in women with low levels of IL‐6 (Figure 3G) (supplementary material, Table S9).

Collectively, this study shows that women carrying the gain‐of‐function variant p.Met1154Val in NLRP1 have a lower risk of malaria during pregnancy and placental malaria in an endemic area. Moreover, we confirm the crucial role of IL‐1β in controlling parasitemia but also as a risk factor for the development of PM.

## Discussion

Our study has led to three main findings: it corroborates the role of *NLRP1* and inflammasome genetics in pregnancy‐related complications. It also provides the first evidence of an association of the *NLRP1* gene with *Plasmodium* infection. Furthermore, we highlight the dual role of IL‐1β genetics in malaria: although this cytokine supports the control of *Plasmodium*, it may have harmful effects during GM.

Animal models of *Plasmodium*‐associated diseases have emphasized the involvement of other inflammasome sensors, such as NLRP3 and AIM2, in detecting protozoan parasites in infected erythrocytes or hepatocytes [8]. During GM, NLRP3 complex activation has been observed in monocytes/macrophages and trophoblast cells [5].

NLRP1 has been identified as crucial in *Toxoplasma gondii* infection in both Lewis rats [15, 17] and humans [14, 16], indicating a potential role in the innate immune response against protozoan parasites. NLRP1 is mainly expressed in keratinocytes and endothelial cells but also in macrophages [30, 33], which are responsible for the uptake of infected erythrocytes and lymphocytes [5, 8]. In the placenta, trophoblasts express the receptor [7], and it appears to be responsible for mediating the oxidative stress response [18, 19].

The exact mechanism for NLRP1 activation in humans remains partially elusive. In the last decade, keynote studies have described two cleavage sites in NLRP1 necessary for activating the correspondent inflammasome. One is localized within the so‐called ‘tripwire’ region at the amino (N‐) terminal portion of the receptor, and the other, an autocleavage site, within the FIIND domain (Phe1212/Ser1213), which is masked by the interaction with dipeptidyl peptidase‐9 (DPP‐9) in the close/inactive conformation of NLRP1. Cleavage of the N‐terminal portion of NLRP1 by specific viral proteases [13] and/or the displacement of DPP‐9 mediated by its chemical inhibitor Val‐boroPro (talabostat) [23, 33] induces liberation of the carboxy‐ (C‐) terminal portion of NLRP1 which includes the CARD domain responsible for the recruitment and activation of caspase‐1. It remains controversial that the two cleavages/signals are needed for activation of NLRP1. The unfolded protein response (UPR)‐mediated pathway and ATF‐4 factor have also been described as upstream factors linked to NLRP1 activation [34]. NLRP1 is also restrained by thioredoxin‐1 (TRX1), and in response to ribotoxic stress, ZAKα and p38 MAP kinase induce phosphorylation of the tripwire linker motif, inducing activation of the NLRP1 inflammasome [35]. Recently, two studies demonstrated that ribotoxic stress induced by *Pseudomonas aeruginosa* and *Corynebacterium diphtheriae* EEF2‐targeting exotoxins leads to NLRP1 inflammasome activation [36, 37].

Therefore, *Plasmodium* infection may induce NLRP1 activation in several ways, including the above‐mentioned UPR [38] or p38 MAPK [39], and further investigations are needed to elucidate the NLRP1–parasite interaction.

p.Met1154Val (rs11651270) is reported to be a gain‐of‐function variant. Differently from p.Leu155His (rs12150220), which is localized within the PYD–NACHT linker region and possibly affected the N‐terminal cleavage site [13], the p.Met1154Val variant is localized within the FIIND domain and is thought to increase the displacement of DPP‐9, favoring activation of the receptor [23, 33].

Of note, the *DPP9* gene was less expressed in *Plasmodium*‐infected monocytes of the Fulani people, an African ethnic group less susceptible to malaria [40], corroborating our findings.

Moreover, the similar frequency of rs11651270 in European and African populations suggests a possible common environmental factor acting across these two continents, such as the malaria that has been endemic in Africa and the Mediterranean region of Europe (up to 50–70 years ago). The other gain‐of‐function variant in *NLRP1*, rs12150220, which was not associated with GM, is not involved in DPP‐9 interaction. Furthermore, it appears to have been fixed at high frequency due to a previous viral epidemic, specifically in the European population (frequency of 50%, compared with 3–19% in non‐European populations) [41] Beyond our initial purpose, we demonstrated that *P. falciparum* iRBCs induce caspase‐1 activation and a lytic cell death compatible with pyroptosis in BeWo trophoblast cells. The observed oligomerization of NLRP1, along with the reduction in caspase‐1 activation and cell death in the presence of the inhibitor ADS‐032, suggests that NLRP1 is involved in inflammasome activation in BeWo cells in response to iRBCs. This is the first evidence of a role of NLRP1 in *Plasmodium* infection. Further investigations are needed to clarify whether iRBCs activate NLRP1 in trophoblasts due to its higher expression level compared with other receptors, such as NLRP3, or if activation depends on upstream pathogenic mechanisms. These preliminary findings support the genetic association data, and appear to be in accordance with recent studies on *T. gondii* infection in trophoblasts [16, 17, 18], suggesting a central role for NLRP1 in the placental response to apicomplexan parasites.

Conflicting data resulted from the genetic association study in PM. On the one hand, our findings revealed the protective effect of the *NLRP1* rs11651270 and *IL1B* rs16944 variants against placental damage. On the other hand, we previously reported that high levels of IL‐1β contribute to PM in our mouse model [5]. This discrepancy may be due to the cell‐specific effect of inflammasome and NLRP1 or to the non‐canonical function eventually played by the receptor. Recent reports showed that *Plasmodium* infection induces ferroptosis, a cell death described in the placenta and correlated with NLRP1 [42], therefore representing another crucial link between NLRP1 and placental malaria. Moreover, functional studies on inflammasome SNVs are rare and normally conducted in peripheral blood mononuclear cells (PBMC) or hyper‐expression cell line models; therefore, our interpretation could be partial. As a proof of concept, while *NLRP1* rs11651270 increases inflammasome activation in PBMCs [43], in the HEK‐293 cell line the same variant increases NLRP1 activation in the context of N‐terminal destabilization, but decreases NLRP1 activation on DPP‐9 inhibition [44]. Moreover, *NLRP1* rs11651270 affects the levels of IL‐6, a cytokine important in malaria, especially in *P. vivax* malaria [45], suggesting that beyond the direct effect of the variant on IL‐1β release and/or pyroptosis, other secondary effects could be hypothesized.

Finally, it is interesting to point out our findings about *NLRP1* and angiogenic factors. Angiogenic dysregulation is involved in placental abnormality, and in gestational malaria [46]. Up to now, no data have been available about the effect of inflammasome genes on angiogenesis. Therefore, our findings linking NLRP1 and VEGFR‐1 may open new interpretations of inflammasome and vessels’ biology, as previously suggested for endothelium [34].

Collectively, this association study shows that women carrying the gain‐of‐function variant p.Met1154Val in NLRP1 have a lower risk of GM. Our findings revealed that this variant significantly reduces critical factors involved in placental inflammation, such as VEGFR‐1 and IL‐6 levels, and placental damage, including syncytial nuclear aggregates and necrosis. To our knowledge, we show, for the first time, that *P. falciparum*‐infected erythrocytes activate the inflammasome in an NLRP1‐ and caspase‐1‐dependent manner.

## Author contributions statement

VNCL designed and performed *in vitro* experiments. JAR determined cytokine and LDH dosage. LGM, ATR and ECR performed DNA isolation and genotyping. GW made the *Plasmodium*‐infected erythrocyte cultures. JGD and CRFM designed and organized the case/control cohort. AP designed the study and wrote the manuscript.

## Supporting information


**Figure S1.** Epistasis analysis for parasitemia
**Figure S2**. Priming does not affect BeWo activation
**Table S1**. Taqman assays
**Table S2**. Frequency of inflammasome variants in the gestational malaria (GM) cohort
**Table S3**. Confounding variables (GLM analysis)
**Table S4**. Detailed results of case/control association analysis
**Table S5**. Detailed results of *NLRP1* p.Met1184Val (rs11651270) analysis in malaria infection
**Table S6**. Main results of the distribution of SNVs in the PM group
**Table S7**. Detailed results of analysis of the SNV distribution according to placental factors
**Table S8**. Correlation analysis of IL‐1β with placental blood factors
**Table S9**. Placental blood factors’ association analysis

## Data Availability

The data that support the findings of this study are available from the corresponding author upon reasonable request.

## References

[1] Bauserman M , Conroy AL , North K , *et al*. An overview of malaria in pregnancy. Semin Perinatol 2019; 43: 282–290. 30979598 10.1053/j.semperi.2019.03.018 PMC7895297

[2] Rogerson SJ , Hviid L , Duffy PE , *et al*. Malaria in pregnancy: pathogenesis and immunity. Lancet Infect Dis 2007; 7: 105–117. 17251081 10.1016/S1473-3099(07)70022-1

[3] Sánchez KE , Spencer LM . Pregnancy‐associated malaria: effects of cytokine and chemokine expression. Travel Med Infect Dis 2022; 47: 102282. 35314344 10.1016/j.tmaid.2022.102282

[4] Bôtto‐Menezes C , Dos Silva Santos MC , Lopes Simplício J , *et al*. *Plasmodium vivax* malaria in pregnant women in the Brazilian Amazon and the risk factors associated with prematurity and low birth weight: a descriptive study. PLoS One 2015; 10: e0144399. 26675007 10.1371/journal.pone.0144399 PMC4687654

[5] Reis AS , Barboza R , Murillo O , *et al*. Inflammasome activation and IL‐1 signaling during placental malaria induce poor pregnancy outcomes. Sci Adv 2020; 6: eaax6346. 32181339 10.1126/sciadv.aax6346 PMC7056302

[6] Mulla MJ , Myrtolli K , Potter J , *et al*. Uric acid induces trophoblast IL‐1β production via the inflammasome: implications for the pathogenesis of preeclampsia. Am J Reprod Immunol 2011; 65: 542–548. 21352397 10.1111/j.1600-0897.2010.00960.x PMC3114103

[7] Pontillo A , Girardelli M , Agostinis , *et al*. Bacterial LPS differently modulates inflammasome gene expression and IL‐1β secretion in trophoblast cells, decidual stromal cells, and decidual endothelial cells. Reprod Sci 2013; 20: 563–566. 23184659 10.1177/1933719112459240

[8] de Carvalho RVH , Zamboni DS . Inflammasome activation in response to intracellular protozoan parasites. Trends Parasitol 2020; 36: 459–472. 32298633 10.1016/j.pt.2020.02.006

[9] Shio MT , Eisenbarth SC , Savaria M , *et al*. Malarial hemozoin activates the NLRP3 inflammasome through Lyn and Syk kinases. PLoS Pathog 2009; 5: e1000559. 19696895 10.1371/journal.ppat.1000559 PMC2722371

[10] Kalantari P , DeOliveira RB , Chan J , *et al*. Dual engagement of the NLRP3 and AIM2 inflammasomes by *Plasmodium*‐derived hemozoin and DNA during malaria. Cell Rep 2014; 6: 196–210. 24388751 10.1016/j.celrep.2013.12.014 PMC4105362

[11] Fernandes FP , Leal VNC , Souza de Lima D , *et al*. Inflammasome genetics and complex diseases: a comprehensive review. Eur J Hum Genet 2020; 28: 1307–1321. 32499599 10.1038/s41431-020-0631-y PMC7608315

[12] Santos MLS , Reis EC , Bricher PN , *et al*. Contribution of inflammasome genetics in *Plasmodium vivax* malaria. Infect Genet Evol 2016; 40: 162–166. 26946405 10.1016/j.meegid.2016.02.038

[13] Castro LK , Daugherty MD . Tripping the wire: sensing of viral protease activity by CARD8 and NLRP1 inflammasomes. Curr Opin Immunol 2023; 83: 102354. 37311351 10.1016/j.coi.2023.102354 PMC10528193

[14] Witola WH , Mui E , Hargrave A , *et al*. NALP1 influences susceptibility to human congenital toxoplasmosis, proinflammatory cytokine response, and fate of *toxoplasma gondii*‐infected monocytic cells. Infect Immun 2011; 79: 756–766. 21098108 10.1128/IAI.00898-10 PMC3028851

[15] Ewald SE , Chavarria‐Smith J , Boothroyd JC . NLRP1 is an inflammasome sensor for *Toxoplasma gondii* . Infect Immun 2014; 82: 460–468. 24218483 10.1128/IAI.01170-13 PMC3911858

[16] Quan JH , Gao FF , Ma TZ , *et al*. *Toxoplasma gondii* induces Pyroptosis pyroptosis in human placental trophoblast and amniotic cells by inducing ROS production and activation of cathepsin B and NLRP1/NLRP3/NLRC4/AIM2 inflammasome. Am J Pathol 2023; 193: 2047–2065. 37741453 10.1016/j.ajpath.2023.08.016

[17] Wang Y , Hollingsworth LR , Sangaré LO , *et al*. Host E3 ubiquitin ligase ITCH mediates *Toxoplasma gondii* effector GRA35‐triggered NLRP1 inflammasome activation and cell‐autonomous immunity. MBio 2024; 20: e0330223. 10.1128/mbio.03302-23 PMC10936166 38376248

[18] Li M , Sun T , Wu X , *et al*. Autophagy in the HTR‐8/SVneo cell oxidative stress model is associated with the NLRP1 inflammasome. Oxid Med Cell Longev 2021; 2021: 2353504. 33854691 10.1155/2021/2353504 PMC8019638

[19] Li M , Wu X , An P , *et al*. Effects of resveratrol on autophagy and the expression of inflammasomes in a placental trophoblast oxidative stress model. Life Sci 2020; 256: 117890. 32497634 10.1016/j.lfs.2020.117890

[20] Pontillo A , Reis EC , Bricher PN , *et al*. *NLRP1* L155H polymorphism is a risk factor for preeclampsia development. Am J Reprod Immunol 2015; 73: 577–581. 25556596 10.1111/aji.12353

[21] Ismail MR , Ordi J , Menendez C , *et al*. Placental pathology in malaria: a histological, immunohistochemical, and quantitative study. Hum Pathol 2000; 31: 85–93. 10665918 10.1016/s0046-8177(00)80203-8

[22] Dombrowski JG , Souza RM , Lima FA , *et al*. Association of malaria infection during pregnancy with head circumference of newborns in the Brazilian Amazon. JAMA Netw Open 2019; 2: e193300. 31050780 10.1001/jamanetworkopen.2019.3300 PMC6503507

[23] Huang M , Zhang X , Toh GA , *et al*. Structural and biochemical mechanisms of NLRP1 inhibition by DPP9. Nature 2021; 592: 773–777. 33731929 10.1038/s41586-021-03320-w PMC8081665

[24] Coll RC , Robertson AA , Chae JJ , *et al*. A small‐molecule inhibitor of the NLRP3 inflammasome for the treatment of inflammatory diseases. Nat Med 2015; 21: 248–255. 25686105 10.1038/nm.3806 PMC4392179

[25] Docherty CA , Fernando AJ , Rosli S , *et al*. A novel dual NLRP1 and NLRP3 inflammasome inhibitor for the treatment of inflammatory diseases. Clin Transl Immunol 2023; 12: e1455. 10.1002/cti2.1455 PMC10288073 37360982

[26] Leal VNC , Roa MEGV , Cantoni JS , *et al*. Integrated genetic and cellular analysis reveals NLRP1 activation in CD4+ T lymphocytes during chronic HIV infection. Immunol Invest 2025; 54: 147–166. 39495019 10.1080/08820139.2024.2419940

[27] González JR , Armengol L , Solé X , *et al*. SNPassoc: an R package to perform whole genome association studies. Bioinformatics 2007; 23: 644–645. 17267436 10.1093/bioinformatics/btm025

[28] Oliveira A , Dinis‐Oliveira RJ , Nogueira A , *et al*. Interleukin‐1β genotype and circulating levels in cancer patients: metastatic status and pain perception. Clin Biochem 2014; 47: 1209–1213. 24747159 10.1016/j.clinbiochem.2014.04.009

[29] Hall SK , Perregaux DG , Gabel CA , *et al*. Correlation of polymorphic variation in the promoter region of the interleukin‐1 beta gene with secretion of interleukin‐1 beta protein. Arthritis Rheum 2004; 50: 1976–1983. 15188375 10.1002/art.20310

[30] Yu CH , Moecking J , Geyer M , *et al*. Mechanisms of NLRP1‐mediated autoinflammatory disease in humans and mice. J Mol Biol 2018; 430: 142–152. 28733143 10.1016/j.jmb.2017.07.012

[31] Sun Y , Lv D , Xie Y , *et al*. PINK1‐mediated mitophagy induction protects against preeclampsia by decreasing ROS and trophoblast pyroptosis. Placenta 2023; 143: 1–11. 37788592 10.1016/j.placenta.2023.09.010

[32] Zhang H , Jiang N , Xu M , *et al*. M2 macrophage derived exosomal miR‐20a‐5p ameliorates trophoblast pyroptosis and placental injuries in obstetric antiphospholipid syndrome via the TXNIP/NLRP3 axis. Life Sci 2025; 370: 123561. 40127859 10.1016/j.lfs.2025.123561

[33] Taabazuing CY , Griswold AR , Bachovchin DA . The NLRP1 and CARD8 inflammasomes. Immunol Rev 2020; 297: 13–25. 32558991 10.1111/imr.12884 PMC7483925

[34] D'Osualdo A , Anania VG , Yu K , *et al*. Transcription factor ATF4 induces NLRP1 inflammasome expression during endoplasmic reticulum stress. PLoS One 2015;; 10: e0130635. 26086088 10.1371/journal.pone.0130635 PMC4472728

[35] Zhang Z , Shibata T , Fujimura A , *et al*. Structural basis for thioredoxin‐mediated suppression of NLRP1 inflammasome. Nature 2023; 622: 188–194. 37704723 10.1038/s41586-023-06532-4

[36] Pinilla M , Mazars R , Vergé R , *et al*. EEF2‐inactivating toxins engage the NLRP1 inflammasome and promote epithelial barrier disruption. J Exp Med 2023; 220: e20230104. 37642996 10.1084/jem.20230104 PMC10465324

[37] Robinson KS , Toh GA , Firdaus MJ , *et al*. Diphtheria toxin activates ribotoxic stress and NLRP1 inflammasome‐driven pyroptosis. J Exp Med 2023;; 220: e20230105. 37642997 10.1084/jem.20230105 PMC10465786

[38] Galluzzi L , Diotallevi A , Magnani M . Endoplasmic reticulum stress and unfolded protein response in infection by intracellular parasites. Future Sci OA 2017; 3: FSO198. 28883998 10.4155/fsoa-2017-0020 PMC5583660

[39] Khadjavi A , Valente E , Giribaldi G , *et al*. Involvement of p38 MAPK in haemozoin‐dependent MMP‐9 enhancement in human monocytes. Cell Biochem Funct 2014; 32: 5–15. 23468369 10.1002/cbf.2963

[40] Quin JE , Bujila I , Chérif M , *et al*. Major transcriptional changes observed in the Fulani, an ethnic group less susceptible to malaria. Elife 2017; 6: e29156. 28923166 10.7554/eLife.29156 PMC5629023

[41] Vasseur E , Boniotto M , Patin E , *et al*. The evolutionary landscape of cytosolic microbial sensors in humans. Am J Hum Genet 2012; 91: 27–37. 22748209 10.1016/j.ajhg.2012.05.008 PMC3397270

[42] Li M , Gao S , Kang M , *et al*. The ferroptosis‐NLRP1 inflammasome: the vicious cycle of an adverse pregnancy. Front Cell Dev Biol 2021; 9: 707959. 34490257 10.3389/fcell.2021.707959 PMC8417576

[43] Zhong FL , Robinson K , Teo DET , *et al*. Human DPP9 represses NLRP1 inflammasome and protects against autoinflammatory diseases via both peptidase activity and FIIND domain binding. J Biol Chem 2018; 293: 18864–18878. 30291141 10.1074/jbc.RA118.004350 PMC6295727

[44] Moecking J , Laohamonthonkul P , Chalker K , *et al*. NLRP1 variant M1184V decreases inflammasome activation in the context of DPP9 inhibition and asthma severity. J Allergy Clin Immunol 2021; 147: 2134–2145.e20. 33378691 10.1016/j.jaci.2020.12.636 PMC8168955

[45] da Costa AG , Antonelli LR , Costa PA , *et al*. The robust and modulated biomarker network elicited by the *Plasmodium vivax* infection is mainly mediated by the IL‐6/IL‐10 axis and is associated with the parasite load. J Immunol Res 2014; 2014: 318250. 24741587 10.1155/2014/318250 PMC3987793

[46] Ataíde R , Murillo O , Dombrowski JG , *et al*. Malaria in pregnancy interacts with and alters the angiogenic profiles of the placenta. PLoS Negl Trop Dis 2015; 9: e0003824. 26090803 10.1371/journal.pntd.0003824 PMC4475015

