# Supplementary material for: Impact of NLRP1 Met1154Val and IL1B variants on gestational malaria: an unexplored role of NLRP1 in inflammasome activation by Plasmodium spp
Source: J Pathol. 2025 Sep 9;267(3):304–14. doi: 10.1002/path.6471 (PMC12531125; doi:10.1002/path.6471)
Supplement: Supplementary file 1 — Figure S1. Epistasis analysis for parasitemia Figure S2. Priming does not affect BeWo activation Table S1. Taqman assays Table S2. Frequency of inflammasome variants in the gestational malaria (GM) cohort Table S3. Confounding variables (GLM analysis) Table S4. Detailed results of case/control association analysis Table S5. Detailed results of NLRP1 p.Met1184Val (rs11651270) analysis in malaria infection Table S6. Main results of the distribution of SNVs in the PM group Table S7. Detailed results of analysis of the SNV distribution according to placental factors Table S8. Correlation analysis of IL‐1β with placental blood factors Table S9. Placental blood factors’ association analysis [file PATH-267-304-s001.docx]

**Impact of *NLRP1* Met1154Val and *IL1B* variants on gestational malaria: an unexplored role of NLRP1 in inflammasome activation by *Plasmodium* spp.**

VNC Leal *et al. J Pathol* <https://doi.org/10.1002/path.6471>

**Supplementary Figures S1 and S2**

**Supplementary Tables S1–S9**

Reference numbers refer to the main text list


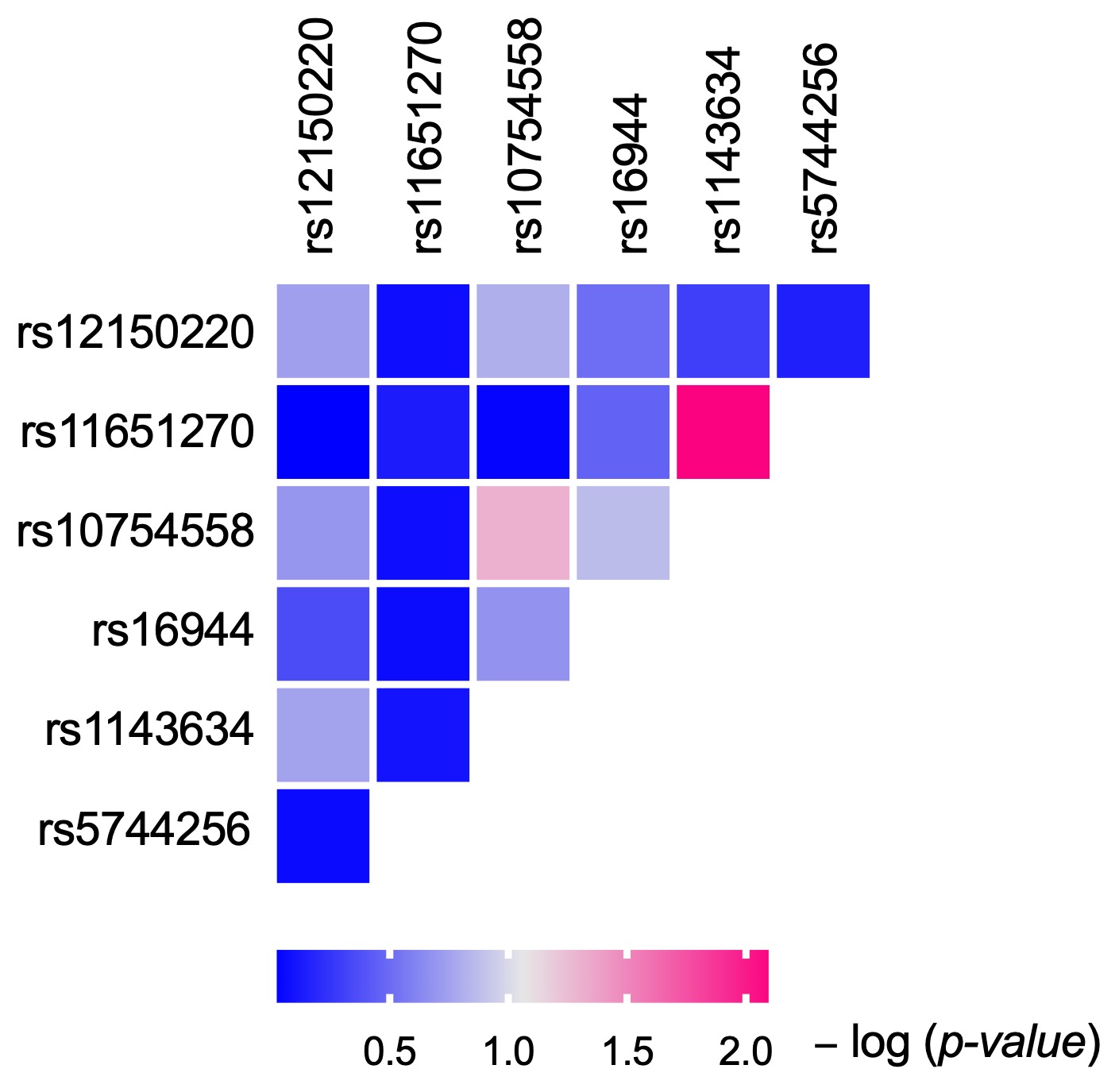


**Figure S1. Epistasis analysis for parasitemia.** Heatmap of epistasis *p* values calculated for SNVs and parasitemia (log parasites/ml) in infected women (GM). Epistasis analysis was performed by use of the ‘epistasis’ function in the SNPassoc package in the R-project [27] to evaluate interactions between two SNPs to understand their joint effects on a phenotype (parasitemia).


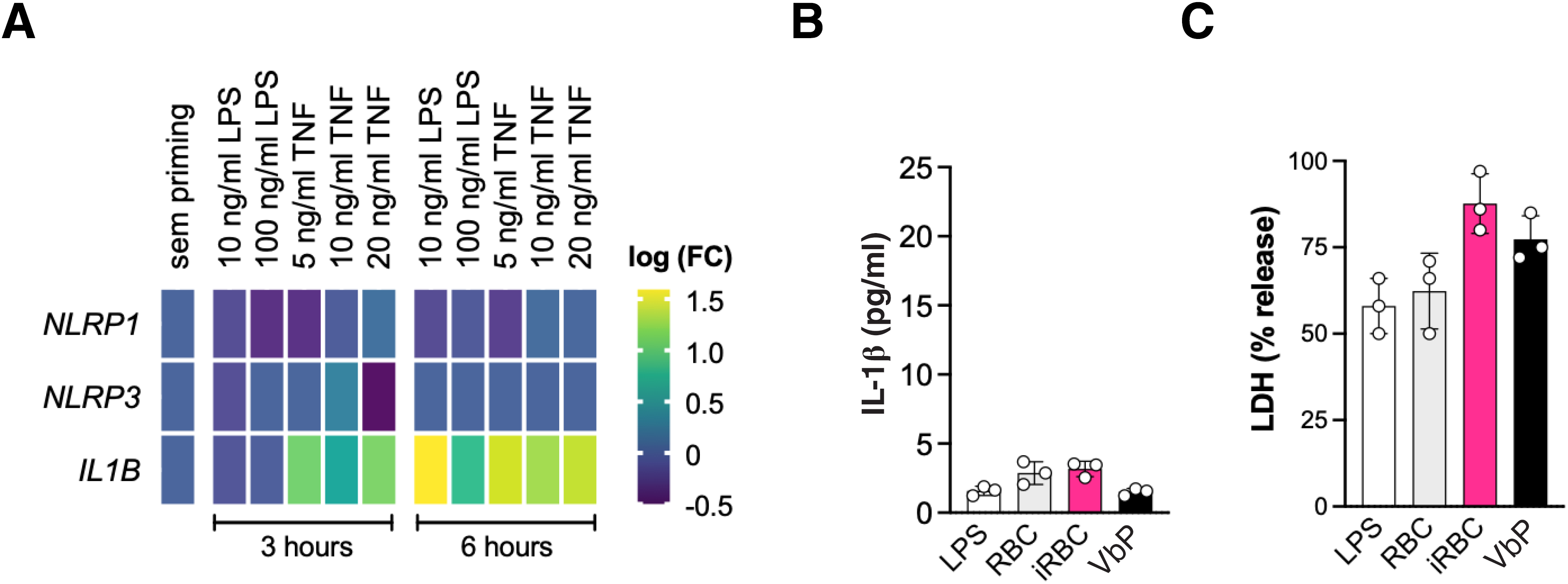


**Figure S2. Priming does not affect BeWo** **activation.** (A) Heatmap of relative expression of *NLRP1*, *NLRP3*, and *IL1B* genes in BeWo cells primed with different concentrations of LPS or TNF. Values are expressed as fold-change (FC) = 2 − exp ∆∆Ct, where ∆Ct was calculated as Ct target gene − Ct GAPDH (reference gene) (B, C) IL-1β and LDH release in BeWo cells primed with LPS, 10 ng/ml, for 6 h and then incubated with RBCs, iRBCs, or Val-boroPro (VbP). The differences were calculated by a one-way ANOVA test followed by a multiple comparisons *post hoc* test.

Table S1. Taqman assays.

| **Gene** | **SNV ID** | **TaqMan Assay ID** | **Change** | **Amino acid** |
| --- | --- | --- | --- | --- |
| *NLRP1* | rs12150220 | C160065310 | chr17:5582047 A>T  c.464T>A | p.Leu155His |
| *NLRP1* | rs11651270 | C3155820010 | chr17:5521757 T>C  c.3550 A>G | p.Met1154Val |
| *NLRP3* | rs10754558 | C2605202810 | chr1:247448734 G>C  c.*230 G>C |  |
| *NLRP3* | rs35829419 | C2564861510 | chr1:247425556 C>A  c.2113 C>A | p.Gln705Lys |
| *IL1B* | rs16944 | C183994310 | chr2:112837290 A>G  g.112837290 A>G |  |
| *IL1B* | rs1143634 | C954651710 | chr2:112832813 G>A  c.315 C>T | p.Phe105= |
| *IL18* | rs5744256 | C289846810 | chr11:112152125 A>G  g.112152125 A>G |  |
| *P2RX7* | rs2230911 | C1585370520 | chr12:121177328 C>G  c.1070 C>G | p.Thr357Ser |
| *DPP9* | rs12610495 | C259662720 | chr19:4717660 A>G  c.-229-19 A>G |  |
| **Gene** |  | **TaqMan Assay ID** |  |  |
| *NLRP1* |  | Hs00248187m1 |  |  |
| *NLRP3* |  | Hs00918082m1 |  |  |
| *IL1B* |  | Hs01555410m1 |  |  |

According to the American Society of Human Genetics (ASHG) and the Human Genome Variation Society (HGVS), the nomenclature for synonymous changes is as follows: p.Phe105Phe or p.Phe105=; however, the HGVS recommends using the = sign instead of repeating the amino acid twice to emphasize that no change occurred.

Table S2. Frequency of inflammasome variants in the gestational malaria (GM) cohort.

| SNV ID | Allele | **MAF** | HWE *p* | **MAF EUR** |  | *p* | **MAF AFR** | *p* | **MAF Mixed** | *p* |
| --- | --- | --- | --- | --- | --- | --- | --- | --- | --- | --- |
| rs12150220 A>T | T | 30.4 | 0.229 | 44 |  | 0.069 | 3 | 0.005 | 23.5 | 0.430 |
| rs11651270 T>C | C | 42 | 0.152 | 46 |  | 0.586 | 48 | 0.405 | 47 | 0.492 |
| rs10754558 C>G | G | 32.6 | 0.627 | 46 |  | 0.068 | 25 | 0.380 | 35.5 | 0.718 |
| rs35829419 C>A | A | 2.8 | 1.000 | 5 |  | 0.821 | 1 | 0.856 | 3 | 0.984 |
| rs16944 G>A | A | 49.6 | 1.000 | 35 |  | 0.070 | 57 | 0.259 | 46 | 0.624 |
| rs1143634 G>A | A | 16.7 | 0.649 | 25 |  | 0.338 | 12 | 0.616 | 18.5 | 0.842 |
| rs5744256 A>G | G | 12.3 | 0.561 | 22 |  | 0.272 | 1 | 0.256 | 11.5 | 0.932 |
| rs2230911 C>G | G | 15.9 | 0.427 | 8 |  | 0.410 | 16 | 0.991 | 12 | 0.678 |
| rs12610495 A>G | G | 20 | 0.626 | 29 |  | 0.285 | 13 | 0.453 | 21 | 0.910 |

SNV ID, single nucleotide variant identification number; MAF, minor allele frequency; HWE, Hardy–Weinberg equilibrium; EUR, European population from the 1000 Genomes Project; AFR, African population from the 1000 Genomes Project; *p*, *χ*^2^ test *p* value.

Table S3. Confounding variables (GLM analysis).

|  | **Estimate** | **Std. error** | ***z* value** | **Pr(>\|*z*\|)** | **Significance** |
| --- | --- | --- | --- | --- | --- |
| **Malaria infection** |  |  |  |  |  |
| Intercept | 0.000137 | 1.86030 | 0.48773 | 3.814 | *** |
| Age | −0.08923 | 0.02058 | −4.336 | 1.45e−05 | *** |
| Parity | 0.33443 | 0.08006 | 4.177 | 2.95e−05 | *** |
| Ethnicity | 0.02125 | 0.44286 | 48 | 0.961732 |  |
| **Placental malaria** |  |  |  |  |  |
| Intercept | 0.35011 | 0.95097 | 0.368 | 0.7128 |  |
| Age | −0.02893 | 0.02983 | −0.970 | 0.3322 |  |
| Parity | −0.03234 | 0.37865 | −0.085 | 0.9319 |  |
| Ethnicity | −0.47728 | 0.70208 | −0.680 | 0.4966 |  |
| *Plasmodium* spp. | −2.76829 | 0.42726 | −6.479 | 9.22e−11 | *** |
| Episodes | 0.33653 | 0.15487 | 2.17 | 0.0298 | * |

**p* < 0.05, ****p* < 0.001.

Table S4. Detailed results of case/control association analysis.

| **SNV ID** | **Genotypes** | **GM**  **(*n* = 282)** | **NI**  **(*n* = 171)** | ***p*** | **adj *p*** |
| --- | --- | --- | --- | --- | --- |
| rs12150220 | A/A | 160 (49.8) | 81 (45.5) | 0.49048 | 0.65372 |
|  | A/T | 129 (40.2) | 77 (43.3) |  |  |
|  | T/T | 32 (10.0) | 20 (11.2) |  |  |
| **rs11651270** | A/A | 119 (39.4) | 46 (26.3) | **0.00120** | **0.00388** |
|  | A/G | 130 (43.0) | 90 (51.4) |  |  |
|  | G/G | 53 (17.5) | 39 (22.3) |  |  |
| rs10754558 | C/C | 142 (44.5) | 78 (43.8) | 0.36163 | 0.56039 |
|  | C/G | 149 (46.7) | 78 (43.8) |  |  |
|  | G/G | 28 (8.8) | 22 (12.4) |  |  |
| rs35829419 | C/C | 54 (98.2) | 28 (93.3) | 0.48229 | 0.29289 |
|  | C/A | 1 (1.8) | 2 ( 6.7) |  |  |
|  | A/A | - | - |  |  |
| rs16944 | A/A | 82 (25.2) | 46 (25.4) | 0.89920 | 0.97825 |
|  | A/G | 164 (50.3) | 88 (48.6) |  |  |
|  | G/G | 80 (24.5) | 47 (26.0) |  |  |
| rs1143634 | C/C | 224 (69.1) | 125 (69.1) | 0.50147 | 0.16176 |
|  | C/T | 92 (28.4) | 48 (26.5) |  |  |
|  | T/T | 8 (2.5) | 8 (4.4) |  |  |
| rs5744256 | A/A | 262 (79.6) | 135 (73.4) | 0.15343 | 0.41254 |
|  | A/G | 62 (18.8) | 44 (23.9) |  |  |
|  | G/G | 5 (1.5) | 5 (2.7) |  |  |
| rs2230911 | C/C | 218 (68.3) | 133 (74.7) | 0.29604 | 0.2702 |
|  | C/G | 94 (29.5) | 42 (23.6) |  |  |
|  | G/G | 7 ( 2.2) | 3 (1.7) |  |  |

*Note*: Statistically significant association is indicated in red text.

SNV ID, single nucleotide variant identification number; GM, pregnant women positive for *Plasmodium* spp. infection [gestational malaria (GM)]; NI, non-infected pregnant women; adj *p*, multivariate analysis *p* value adjusted for confounding variables.

Table S5. Detailed results of *NLRP1* p.Met1184Val (rs11651270) analysis in malaria infection.

| **SNV ID** | **Genotypes** | **All GM**  **(*n* = 282)** | **NI**  **(*n* = 171)** | **adj OR** | **adj *p*** |
| --- | --- | --- | --- | --- | --- |
| rs11651270 | A/A (Met) | 109 (42.4) | 41 (25.8) | Ref | 6 × 10^−4^ |
|  | A/G | 110 (42.8) | 85 (53.5) | 0.47 (0.30–0.73) |  |
|  | G/G (Val) | 38 (14.8) | 33 (20.8) | 0.47 (0.30–0.73) |  |
| **SNV ID** | **Genotypes** | ***Pf* GM**  **(*n*= 103)** | **NI**  **(*n*= 171)** | **adj OR** | **adj *p*** |
| rs11651270 | A/A (Met) | 33 (47.8) | 41 (25.8) | Ref | 7.5 × 10^−4^ |
|  | A/G | 31 (44.9) | 85 (53.5) | 0.35 (0.19–0.65) |  |
|  | G/G (Val) | 5 (7.2) | 33 (20.8) | 0.35 (0.19–0.65) |  |
| **SNV ID** | **Genotypes** | ***Pv* GM**  **(*n* = 157)** | **NI**  **(*n*= 171)** | **adj OR** | **adj *p*** |
| rs11651270 | A/A (Met) | 55 (43.3) | 41 (25.8) | Ref | 0.002 |
|  | A/G | 55 (43.3) | 85 (53.5) | 0.46 (0.28–0.76) |  |
|  | G/G (Val) | 17 (13.4) | 33 (20.8) | 0.46 (0.28–0.76) |  |

SNV ID, single nucleotide variant identification number; All GM, pregnant women positive for *Plasmodium* spp. infection [gestational malaria (GM)]; *Pf* GM, pregnant women positive for *P. falciparum* infection; *Pv* GM, pregnant women positive for *P. vivax* infection; NI, non-infected pregnant women; adj *p*, multivariate analysis *p* value adjusted for confounding variables; adj OR, multivariate analysis odds ratio adjusted for confounding variables.

Table S6. Main results of the distribution of SNVs in the PM group.

| **SNV ID** | **Genotypes** | **GM w/o PM**  **(*n*= 191)** | **PM**  **(*n*= 91)** | **adj OR (95% CI)** | **adj *p*** |
| --- | --- | --- | --- | --- | --- |
| rs1143634 | G/G | 145 (75.9) | 56 (61.1) | Ref | 0.002 |
|  | G/A | 43 (22.5) | 34 (37.8) | 2.84 (1.43−5.64) |  |
|  | A/A | 3 (1.6) | 1 (1.1) | 2.84 (1.43−5.64) |  |
| **SNV combination** | |  |  | **adj OR (95% CI)** | **adj *p*** |
| rs11651270 C/C * rs16944 G/A | |  |  | 0.06 (0.00−0.81) | 0.033 |

SNV ID, single nucleotide variant identification number; GM w/o PM, pregnant women positive for *Plasmodium* spp. infection [gestational malaria (GM)] without PM; PM, pregnant women positive for *Plasmodium* spp*.* infection with PM; adj *p*, multivariate analysis *p* value adjusted for confounding variables; adj OR, multivariate analysis odds ratio adjusted for confounding variables; 95% CI, confidence interval.

Table S7. Detailed results of analysis of the SNV distribution according to placental factors.

| **SNV ID** | **Genotypes** | **GM SNAs**  **(*n*)** | **Mean (SE)** | **Diff**  **(lower–upper)** | **adj *p*** |
| --- | --- | --- | --- | --- | --- |
| rs11651270 | A/A (Met) | 83 | 18.34 (1.08) | Ref | 8.6 × 10 ^−4^ |
|  | A/G | 82 | 15.55 (0.70) | −3.19 (−5.55–0.83) |  |
|  | G/G (Val) | 30 | 13.97 (1.27) |  |  |
| **SNV ID** | **Genotypes** | **GM necrosis**  **(*n*)** | **Mean (SE)** | **Diff**  **(lower–upper)** | **adj *p*** |
| rs11651270 | A/A (Met) | 79 | 8.56 (0.55) | Ref | 0.013 |
|  | A/G | 80 | 7.67 (0.43) | −2.23 (−3.97–0.49) |  |
|  | G/G (Val) | 29 | 5.94 (0.65) |  |  |
| **SNV ID** | **Genotypes** | **GM fibrin**  **(*n*)** | **Mean (SE)** | **Diff**  **(lower–upper)** | **adj *p*** |
| rs1143634 | G/G | 148 | 0.35 (0.00) | Ref | 0.009 |
|  | G/A | 61 | 0.35 (0.02) | −0.20 (−0.32–0.08) |  |
|  | A/A | 4 | 0.15 (0.08) |  |  |
| **SNV ID** | **Genotypes** | **PM**  **CD31^+^ cells**  **(*n*)** | **Mean (SE)** | **Diff**  **(lower–upper)** | **adj *p*** |
| rs16944 | C/C | 21 | 4.22 (0.19) | Ref | 0.005 |
|  | C/T | 47 | 4.11 (0.11) |  |  |
|  | T/T | 14 | 3.6 (0.26) | −0.61 (−1.07–0.15) |  |
| **SNV ID** | **Genotypes** | **PM**  **fibrin(*n*)** | **Mean (SE)** | **Diff**  **(lower–upper)** | **adj *p*** |
| rs1143634 | G/G | 55 | 0.34 (0.02) | Ref | 0.004 |
|  | G/A | 34 | 0.33 (0.02) |  |  |
|  | A/A | 1 | 0 | −0.40 (−0.66–0.13) |  |

SNV ID, single nucleotide variant identification number; GM, gestational malaria; PM, placental malaria; SNAs, syncytial nuclear aggregates; adj *p*, multivariate analysis *p* value adjusted for confounding variables; Diff, difference from reference value; Ref, reference value.

**Table S8.** Correlation analysis of IL-1β with placental blood factors.

|  | **IL1b**  **versus**  **ANG-1** | **IL1b**  **versus**  **ANG-2** | **IL1b**  **versus**  **TIE2** | **IL1b**  **versus**  **VEGFR-1** | **IL1b**  **versus**  **VEGFR-r2** | **IL1b**  **versus**  **VEGF** | **IL1b**  **versus**  **C3a** | **IL1b**  **versus**  **C4a** |  | **IL1b**  **versus**  **C5a** | **IL1b**  **versus**  **TNF** | **IL1b**  **versus**  **IL-6** | **IL1b**  **versus**  **IL-12** | **IL1b**  **versus**  **IL-10** |
| --- | --- | --- | --- | --- | --- | --- | --- | --- | --- | --- | --- | --- | --- | --- |
| **NI** |  |  |  |  |  |  |  |  |  |  |  |  |  |  |
| Spearman *r* | −0.255 | 0.325 | −0.561 | 0.534 | −0.211 | −0.064 | 0.114 | 0.011 |  | 0.346 | 0.595 | 0.622 | 0.445 | 0.455 |
| *p* (two-tailed) | 0.002 | <0.0001 | <0.0001 | <0.0001 | 0.010 | 0.444 | 0.170 | 0.892 |  | <0.0001 | <0.0001 | <0.0001 | <0.0001 | <0.0001 |
| *p* value summary | ** | **** | **** | **** | * | ns | ns | ns |  | **** | **** | **** | **** | **** |
| **GM** |  |  |  |  |  |  |  |  |  |  |  |  |  |  |
| Spearman *r* | −0.228 | 0.258 | −0.413 | 0.524 | −0.203 | −0.050 | 0.054 | −0.122 |  | 0.579 | 0.734 | 0.658 | 0.529 | 0.640 |
| *p* (two-tailed) | 0.001 | 0.000 | <0.0001 | <0.0001 | 0.003 | 0.468 | 0.551 | 0.129 |  | <0.0001 | <0.0001 | <0.0001 | <0.0001 | <0.0001 |
| *p* value summary | *** | *** | **** | **** | ** | ns | ns | ns |  | **** | **** | **** | **** | **** |
| **PM** |  |  |  |  |  |  |  |  |  |  |  |  |  |  |
| Spearman *r* | −0.097 | 0.098 | −0.346 | 0.354 | −0.084 | −0.134 | −0.096 | −0.136 |  | 0.474 | 0.694 | 0.539 | 0.505 | 0.645 |
| *p* (two-tailed) | 0.385 | 0.402 | 0.002 | 0.002 | 0.457 | 0.232 | 0.499 | 0.254 |  | <0.0001 | <0.0001 | <0.0001 | <0.0001 | <0.0001 |
| *p* value summary | ns | ns | ** | ** | ns | ns | ns | ns |  | **** | **** | **** | **** | **** |
| **GM w/o PM** |  |  |  |  |  |  |  |  |  |  |  |  |  |  |
| Spearman *r* | −0.368 | 0.335 | −0.464 | 0.610 | −0.267 | 0.046 | 0.219 | −0.113 |  | 0.591 | 0.770 | 0.705 | 0.562 | 0.643 |
| *p* (two-tailed) | <0.0001 | 0.000 | <0.0001 | <0.0001 | 0.002 | 0.608 | 0.062 | 0.300 |  | <0.0001 | <0.0001 | <0.0001 | <0.0001 | <0.0001 |
| *p* value summary | **** | *** | **** | **** | ** | ns | ns | ns |  | **** | **** | **** | **** | **** |

*Note*: Statistically significant association is indicated in red text.

NI, non-infected pregnant women; GM, gestational malaria; PM, placental malaria; GM w/o PM, infected women without placental malaria; ns, not significant; **p* < 0.05; ***p*< 0.01; ****p* < 0.001; ****p*< 0.0001.

**T****able S9.** Placental blood factors’ association analysis.

| **rs11651270** | ***n*** | **Mean (SE)** | **adj *p*** |
| --- | --- | --- | --- |
| ANG-1 | T/T: 83  T/C: 82  C/C: 33 | 4.18 (0.03)  4.19 (0.03)  4.30 (0.05) | 0.015 |
| VEGFR1 | T/T: 73  T/C: 72  C/C: 30 | 4.74 (0.06)  4.88 (0.06)  4.49 (0.10) | 0.002 |
| IL-6 | T/T: 81  T/C: 82  C/C: 33 | 1.88 (0.07)  2.01 (0.07)  1.67 (0.10) | 0.005 |

*n*, number of individuals carrying a genotype; SE, standard error; adj *p*, multivariate analysis *p* value adjusted for confounding variables.
